# Supplementary material for: Improved systemic AAV gene therapy with a neurotrophic capsid in Niemann–Pick disease type C1 mice
Source: Life Sci Alliance. 2021 Aug 18;4(10):e202101040. doi: 10.26508/lsa.202101040 (PMC8380657; doi:10.26508/lsa.202101040)
Supplement: Supplementary file 1 [file LSA-2021-01040_TableS1.docx]

**Supplemental Tables**

**Supplemental Table 1.** Phenotype Scores and Balance Beam: Two-way ANOVA
