# Supplementary material for: Improved systemic AAV gene therapy with a neurotrophic capsid in Niemann–Pick disease type C1 mice
Source: Life Sci Alliance. 2021 Aug 18;4(10):e202101040. doi: 10.26508/lsa.202101040 (PMC8380657; doi:10.26508/lsa.202101040)
Supplement: Supplementary file 2 [file LSA-2021-01040_TableS2.docx]

**Supplemental Table 2.** Genotype, sample size, and gender of mice used in study

| Study group | Age at sacrifice | Sample size |
| --- | --- | --- |
| *Npc1^m1N/m1N^* saline | 9 weeks / End stage disease | 4M, 1F / 2M, 6F |
| *Npc1^m1N/m1N^* AAV9-*NPC1* | 9 weeks / End stage disease | 3M, 4F / 5M, 4F |
| *Npc1^m1N/m1N^* AAV-PHP.B-*NPC1* | 9 weeks / End stage disease | 2M, 2F / 4M, 5F |
| *Npc1^+/+^* AAV-PHP.B-*NPC1* | 9 weeks | 3M, 4F |
| *Npc1^+/+^* no treatment | 9 weeks / End of study | 2M, 3F / 3M, 5F |
| *Npc1^m1N/m1N^* AAV9-GFP | 9 weeks | 2M, 3F |
| *Npc1^m1N/m1N^* AAV-PHP.B-GFP | 9 weeks | 2M, 2F |
| *Npc1^+/+^* AAV9-GFP | 9 weeks | 2M, 3F |
| *Npc1^+/+^* AAV-PHP.B-GFP | 9 weeks | 2M, 4F |
