# Supplementary material for: Improved systemic AAV gene therapy with a neurotrophic capsid in Niemann–Pick disease type C1 mice
Source: Life Sci Alliance. 2021 Aug 18;4(10):e202101040. doi: 10.26508/lsa.202101040 (PMC8380657; doi:10.26508/lsa.202101040)

Full unedited gels for Figure 4

Please see green outlines for cerebrum and blue outlines for liver to denote portion of gels used in Figure 4. Each gel was co-labeled with NPC1 and α-Tubulin followed by different secondary antibodies against each primary.

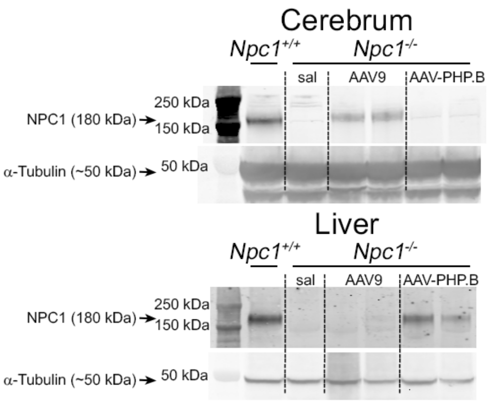

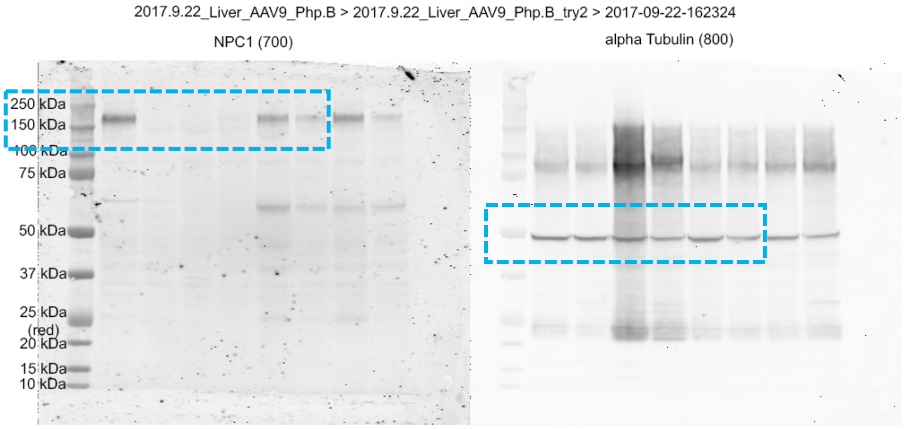

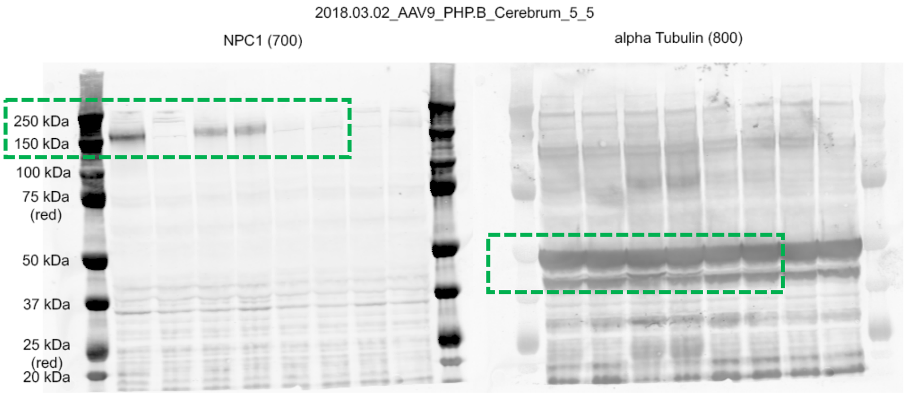

Supplement: Supplementary file 5 [file LSA-2021-01040_SdataF4.pdf]
