# Supplementary material for: Improved systemic AAV gene therapy with a neurotrophic capsid in Niemann–Pick disease type C1 mice
Source: Life Sci Alliance. 2021 Aug 18;4(10):e202101040. doi: 10.26508/lsa.202101040 (PMC8380657; doi:10.26508/lsa.202101040)
Supplement: Supplementary file 6 [file LSA-2021-01040_TableS3.docx]

**Supplemental Table 3.** Antibodies and dilutions for immunofluorescent staining

| Primary Antibody | Company | Catalog # | Dilution |
| --- | --- | --- | --- |
| anti-calbindin | Sigma Aldrich | C9848 | 1:750 |
| anti-GFAP (glial fibrillary astrocyte protein) | Sigma Aldrich | G3893 | 1:1000 |
| anti-IBA1 | Wako Chemicals | 019-19741 | 1:300 |
| anti-macrosialin (CD68) | AbDSerotec | MCA1957 | 1:1000 |
|  |  |  |  |
| Secondary Antibody |  |  |  |
| AlexaFluor 488 (rat IgG, mouse IgG, rabbit IgG) | ThermoFisher Invitrogen | A11006, A11029, A11034 | 1:350 |
| AlexaFluor 594 (rat IgG, mouse IgG, rabbit IgG) | ThermoFisher Invitrogen | A11007, A11005, A11037 | 1:350 |
